# Modulation of PPAR-**γ** by Nutraceutics as Complementary Treatment for Obesity-Related Disorders and Inflammatory Diseases

**DOI:** 10.1155/2012/318613

**Published:** 2012-11-28

**Authors:** D. Ortuño Sahagún, A. L. Márquez-Aguirre, S. Quintero-Fabián, R. I. López-Roa, A. E. Rojas-Mayorquín

**Affiliations:** ^1^Laboratorio de Desarrollo y Regeneración Neural, Instituto de Neurobiología, Departamento de Biología Celular y Molecular, CUCBA, Universidad de Guadalajara, camino Ing. R. Padilla Sánchez 2100, Las Agujas, 44600 Zapopan JAL, Mexico; ^2^Unidad de Biotecnología Médica y Farmacéutica, Centro de Investigación y Asistencia en Tecnología y Diseño del Estado de Jalisco A.C., 44270 Guadalajara, JAL, Mexico; ^3^Departamento de Farmacobiología, CUCEI, Universidad de Guadalajara, Boulevard Marcelino García Barragán, 44430 Tlaquepaque, JAL, Mexico; ^4^Departamento de Ciencias Ambientales, Instituto de Neurociencias, CUCBA, Universidad de Guadalajara, 45100, JAL, Mexico; ^5^Departamento de Investigación Básica, Instituto Nacional de Geriatría (INGER), Periférico Sur No. 2767, Col, San Jerónimo Lídice, Delegación Magdalena Contreras 10200, México DF, Mexico

## Abstract

A direct correlation between adequate nutrition and health is a universally accepted truth. The Western lifestyle, with a high intake of simple sugars, saturated fat, and physical inactivity, promotes pathologic conditions. The main adverse consequences range from cardiovascular disease, type 2 diabetes, and metabolic syndrome to several cancers. Dietary components influence tissue homeostasis in multiple ways and many different functional foods have been associated with various health benefits when consumed. Natural products are an important and promising source for drug discovery. Many anti-inflammatory natural products activate peroxisome proliferator-activated receptors (PPAR); therefore, compounds that activate or modulate PPAR-gamma (PPAR-**γ**) may help to fight all of these pathological conditions. Consequently, the discovery and optimization of novel PPAR-**γ** agonists and modulators that would display reduced side effects is of great interest. In this paper, we present some of the main naturally derived products studied that exert an influence on metabolism through the activation or modulation of PPAR-**γ**, and we also present PPAR-**γ**-related diseases that can be complementarily treated with nutraceutics from functional foods.

## 1. Introduction

Peroxisome proliferator-activated receptors (PPAR) are ligand-activated transcription factors belonging to the nuclear receptor superfamily of regulatory factors. They exercise homeostatic functions in the intestine at the interface between nutrient metabolism and immunity, and their main function is related with the regulation of genes involved in glucose and lipid metabolism [1–4].

In mammals, three isoforms have been identified (*α*, *β*/*δ*, and *γ*), which are encoded by separate genes. All PPAR isotypes form a heterodimeric complex with the retinoid X receptor (RXR), and the complex binds to the PPAR response element, which functions as the central regulator of cellular differentiation [5], apoptosis [6], inflammatory responses [7, 8], lipid metabolism, and metabolic disease [9]. Also, the farnesoid X receptor (FXR) is involved in adipogenesis, adipocyte differentiation, and lipid storage, increasing adiponectin through a mechanism partially mediated by PPAR-gamma (PPAR-*γ*) [10, 11].

PPAR-*γ* is expressed generally in multiple tissues, such as lung, breast, ovary, placenta, and colon. It promotes cell differentiation, particularly as a potent master gene, as an inducer and regulator of adipocyte growth and differentiation, promoting their transition from small, quiescent adipocytes to large, activated adipocytes [12, 13] by a transcriptional cascade that controls the expression of a number of genes that are essential in lipid accumulation in adipocytes during the differentiation and also in mature adipocytes [13–15]. Moreover, it is a key gene involved in the control of hepatic peroxisomal *β*-oxidation of fatty acids [16]. Additionally, PPAR-*γ* has been considered as a molecular target for cancer chemoprevention [17, 18]. In addition, it possesses anti-inflammatory effects and increases insulin sensitivity [19]. More recently, PPAR-*γ* has been shown to be involved also in the regulation of genes contributing to hypertension and atherosclerosis [20].

Adipose tissue is an important source of hormones and cytokines [21]. Therefore, selective PPAR-*γ* agonists exert anti-inflammatory effects while regulating major metabolic pathways in abdominal fat [22]. However, the use of PPAR-*γ* ligands is associated with an increased risk of cardiovascular ischemic events, as in the case of rosiglitazone [23]. In addition, other compounds that exert different effects on PPAR-*γ*, such as tri-methylbenzidine trinitrobenzene sulfonic acid (TNBS), which induced acute inflammation in the mesenteric tract, appear to support a role for this nuclear receptor in promoting the acquisition of a proinflammatory phenotype by mesenteric adipocytes [24].

Despite the fact that PPAR-*γ* activators are currently used as insulin sensitizers to combat type 2 diabetes and metabolic syndrome [25], PPAR-*γ* agonists in clinical use, represented by thiazolidinediones (TZD), have serious side effects such as weight gain, increased bone fracture, fluid retention, and heart failure [26]. Conversely, natural products constitute an important and promising source for drug discovery [27]. Many anti-inflammatory natural products, especially dietary lipids such as linoleic acids, activate PPAR [28].

One of the main problems of using nutraceuticals for experimental research is that they are derived from natural sources, such as plant extracts and microbial products; therefore, in some cases, the active compounds that exert the biological action are not yet completely identified. Moreover, in many cases, the biological action is reduced, or even modified, when the compounds are fractionated or isolated; this is one of the major limitations of nutraceutical experimentation. Clearly, the whole comprises more than simple addition of the components, and this idea also applies to the use of nutraceuticals as dietary complements. In addition, the specific susceptibility, such as the particular physiologic and genomic characteristics of each individual, renders the action of nutraceuticals more complex because this generally implies a balance among many different elements, such as metabolic state, physiological performance, gene expression profiles, and, of course, the composition of the nutritional complements. Additionally, in many cases, the experimental information available remains scarce and in many respects also preliminary, most probably due to the difficulty represented in obtaining active extracts whose composition is not completely established.

In this context, we review diverse nutraceutics that are reported to be able to modulate PPAR-*γ* expression or action and that could in turn be employed as complementary treatment for obesity-related disorders and some inflammatory diseases, reducing the side effects exhibited by commonly used pharmacological drugs.

### 1.1. Diet and Lifestyle

A direct correlation between adequate nutrition and health is a universally accepted truth; “we are what we eat.” Geopolitical diversity, modern science, and economic changes have resulted in the development of new social food habits. Continued changes in the processing technologies to meet consumer preferences and lifestyle changes have caused the present state of increased caloric intake, sedentary habits, overconsumption of high energy foods due to increased portion sizes, and low intake of functional foods, resulting in a significant increase in the prevalence of several chronic degenerative diseases, such as type 2 diabetes, cardiovascular diseases, neurodegenerative diseases, and inflammatory diseases [29].

Worldwide, over 1 billion adults and 10% of children can be classified as overweight or obese [30]. Their average life expectancy is already diminished, and main adverse consequences range from cardiovascular disease, type 2 diabetes, metabolic syndrome, and several cancers, which are currently engaged in a devastating epidemic spread throughout the world. Compounds that activate or modulate PPAR-*γ* may aid in fighting all of these pathological conditions [25].

### 1.2. Functional Foods

The European Commission's Concerted Action on Functional Food Science in Europe (FuFoSE), coordinated by International Life Science Institute (ILSI) Europe, described functional foods as follows:

*“A food can be regarded as ‘functional' if it is satisfactorily demonstrated to affect beneficially one or more target functions in the body, beyond adequate nutritional effects, in a way that is relevant to either an improved state of health and well-being and/or reduction of risk of disease. Functional foods must remain foods and they must demonstrate their effects in amounts that can normally be expected to be consumed in the diet: they are not pills or capsules, but part of a normal food pattern.” *[31, 32]*.*

Functional foods contain bioactive substances, nutraceutics, which can be classified as micronutrients (vitamins and fatty acids) and nonnutrients (phytochemicals and probiotics) (Table 1). These components, with a wide range of chemical structures and functionality, provide different beneficial effects beyond simple nutrition, resulting in improved health. Fruits and vegetables are rich sources of a wide range of vital micronutrients as follows: vitamins (provitamin A, carotenoids, vitamin C, and folate), phytochemicals (nonprovitamin A carotenoids and polyphenols), and fiber (a high proportion of nondigestible carbohydrate polymers such as cellulose, pectin, and fructans). The influence of nondigestible carbohydrate polymers (prebiotics) on intestinal health, through viscosity, modification of foods during their transition through the gastrointestinal tract, immunity modulation, prevention of inflammation, and the maintenance of an ideal population of microbiotics (probiotics) is continuously being unraveled, whereas secondary plant products such as carotenoids, polyphenols, sulforaphanes, indoles, and essential oils, in conjunction with a milieu of polymeric substances from fruits and vegetables, enhance the healthfulness of foods [33].

Some of these nutraceutics have been reported to be able to modulate PPAR-*γ* expression or action and could in turn be used as complementary treatment for obesity-related disorders and inflammatory diseases. In Table 2, we summarize some of the effects exerted on PPAR-*γ* by nutraceutics.

## 2. Micronutrients

According to the World Health Organization (WHO), certain nutrients are called *micronutrients* because they are needed only in minuscule amounts; these substances are the “magic wands” that enable the body to produce enzymes, hormones, and other substances essential for proper growth and development.

### 2.1. Beta-Carotene, Vitamin A, and Its Derivatives

In mammals, beta-carotene (BC) is the natural precursor for apocarotenoid molecules including retinoids (vitamin A and its derivatives) [34]. There is increasing evidence of an impact of BC, vitamin A, and its derivatives as signaling molecules that can influence adipocyte physiology by acting on parameters related with adiposity in humans [35]. Circulating BC levels are inversely correlated with the risk of human type 2 diabetes [36–39]. Additionally, reduced plasma levels of carotenoids, including BC, are commonly found in obese children [40].

An important metabolic linkage is carried out by the BC 15,15′-monooxygenase (*Bcmo1*), which converts BC into all-*trans*-retinal and is the primary enzyme for retinoid production [41]. *Bcmo1* knockout mice are highly susceptible to high fat diet-induced obesity and show increased expression of PPAR-*γ*-regulated genes in fat depots [41]. *Bcmo1* gene expression is under the control of PPAR-*γ* [42, 43] and is induced during adipocyte differentiation [44]. The primary BC cleavage product, retinaldehyde, has been shown to inhibit PPAR-*γ* activity both in adipocyte cell cultures and mouse models [45]. Furthermore, evidence has been provided that *Bcmo1* plays an important role in Retinoic acid (RA) production and in RA receptor (RAR) signaling in adipocytes [44].

Additionally, BC-derived long-chain apocarotenoids, such as *β*-apo-149-carotenal, can inhibit PPAR-*γ* activity and adipogenesis in cell culture [46]. Moreover, *β*-13-apocarotenone has been shown to inhibit retinoid X receptor alpha (RXR*α*) activity [64]. Thus, reduction of PPAR-*γ* activity and downregulation of its target genes likely explain the reduced adiposity of WT mice upon BC supplementation.

Therefore, BC plays an important role in the control of body adiposity in mice and *Bcmo1* is a critical molecular player for the regulation of PPAR-*γ* activity in adipocytes and also comprised a key component for crosstalk between RAR- and PPAR-*γ*-signaling pathways in control of body adiposity [65]. However, the complete specific mechanisms remain to be elucidated. Ongoing studies will offer new insights into adipose physiology and the mechanisms for the regulation of glucose and lipid metabolism by retinoids. These studies will contribute to further understanding of the factors important for adipose tissue development and may lead to future therapeutic interventions. These may include the development of small molecules that directly target adipose tissue development.

### 2.2. Vitamin E: Alpha-Tocopherols and Tocotrienols

Vitamin E is a fat-soluble vitamin that is essential for humans [66]. The vitamin E family comprises eight lipophilic, naturally occurring compounds that include four tocopherols (with a saturated phytyl tail) and four tocotrienols (with an unsaturated isoprenoid side chain) designated as *α*, *β*, *γ*, and *δ* [67]. The *α*- and *γ*-tocopherols are the most common dietary tocopherols due to their high amounts in commercially produced vegetable oils such as soybean, corn, and cottonseed [68, 69]. Alpha tocopherol has been considered the classic “vitamin E” because it is the major tocopherol found in plasma and tissues [69].

Both *α*- and *γ*-tocopherol have been shown to activate PPAR-*γ* expression and transactivation in colon cancer cells, and *γ*-tocopherol is a better modulator of PPAR-*γ* expression than is *α*-tocopherol [47, 48]. Analogs of PPAR-*γ* ligands, *α*-, *γ*-, and mixed tocopherols, are important for the inhibition of cancer in animals [17, 18, 70, 71], suggesting that *γ*- and *δ*-tocopherols should be considered as anticancerogenic nutraceutics for studies in humans.

Tocotrienols are naturally occurring compounds that appear to be nontoxic; therefore, they are used as dietary supplements in some studies, with the ultimate goal of preventing some of the damage attributable to dysregulated inflammatory responses associated with aging. A recent evaluation of the anti-inflammatory properties of dietary supplementation with quercetin and *δ*-tocotrienol, two naturally occurring proteasome inhibitors, *in vivo* in mice [72] and in chickens [73], demonstrate a reduction in serum tumor necrosis factor-alpha (TNF-*α*) and nitric oxide (NO) levels.

Additionally, findings from various studies suggested that tocotrienols exert a direct effect on lipidic metabolism, with a hypocholesterolemic or antiatherogenic effect on humans, rats, and mice. *In vitro* studies demonstrated that tocotrienols act as the 3-hydroxy-3-methyl-glutaryl-CoA reductase (HMGCR) inhibitor and consequently reduce cholesterol synthesis. For example, oral administration of a tocotrienol-rich fraction (TRF) of palm oil, which contains *γ*-tocotrienol, decreases body fat levels in rats. Additionally, in an *in vitro* study, TRF suppressed adipocyte differentiation and Akt phosphorylation in 3T3-L1 preadipocytes, through the suppression of the insulin-induced PPAR-*γ* mRNA expression [74].

These data show the possibility that tocotrienol could be an antiadipogenic vitamin, similar to vitamin A, with regard to nutrient-mediated regulation of body fat through its effects on differentiation. However, further study is required to determine whether tocotrienol could effectively promote the loss of body fat in humans.

### 2.3. Retinoic Acid and 1,25-Dihydroxy Vitamin D3 (1,25 (OH)2 D3)

Retinoic acid (RA) influences adipocyte differentiation [75, 76] and fat deposition [77] and the expression of adipokines such as leptin, resistin, and the serum retinol-binding protein [78–81]. Part of these effects is mediated via RAR [82], which can interfere with the activity of PPAR-*γ* [76, 77]. In addition, RA may influence PPAR-mediated responses by activating the RXR moiety of permissive PPAR:RXR heterodimers [83] and, possibly, by serving as an agonist of PPAR-*β*/*δ* [49, 84].

RA and the 1,25-dihydroxy vitamin D3 (1,25 (OH)2 D3) inhibited adipocyte differentiation of 3T3-L1 preadipocytes by repressing the upregulated protein expression of PPAR-*γ*2 [85]. The active form of vitamin D, (1,25 (OH)2 D3), inhibits adipogenesis in the bone marrow of SAM-P/6 mice and is associated with reduction in PPAR-*γ*2 expression [86].

### 2.4. N3 Polyunsaturated Fatty Acids (PUFAs)

#### 2.4.1. PUFAs-Omega-3 Fatty Acids: DHA/EPA from Fish Oil

The beneficial effect of fish oil (FO) has been demonstrated in several human diseases, including cardiovascular diseases [87], autoimmune inflammatory diseases, rheumatoid arthritis, inflammatory bowel disease, osteoporosis [88], sepsis, vascular compliance, blood pressure [89], and diabetes [90], among other pathologies. FO has been shown to have beneficial effects on glucose and lipid metabolism in studies with rodents, to improve insulin sensitivity [91, 92], to reduce triglycerides [93], and to improve bone mineral density (BMD) in ovariectomized mice [88].

Along with lowering of plasma triglycerides, FO also reduces blood pressure, inflammation, thrombosis, and arrhythmia, contributing to its role in lowering risk of cardiovascular disease and diabetes (reviewed in [50, 94]). In addition, FO supplementation increases adiponectin levels and up-regulates PPAR-*γ* [51, 95].

The n-3 polyunsaturated fatty acids (PUFAs) of FO, eicosapentaenoic acid (EPA) and docosahexaenoic acid (DHA), are endogenous ligands for PPAR; therefore, they mediate the insulin-sensitizing, lipid-lowering, and anti-inflammatory properties of FO [96]. Not only the n-3 fatty acids, but also their metabolites are potent PPAR-*γ* agonists [97]. The interaction between FO and PPAR plays a critical role in regulating glucose and lipid metabolism in liver (reviewed in [98, 99]) and in adipose tissue (reviewed in [96]). Thus, FO mediates its beneficial effects on metabolic syndrome by improving adipose tissue storage and secretory functions and by reducing inflammation.

Finally, it has been hypothesized that derivatives from EPA and DHA (from FO) are stronger PPAR agonists than their parent compounds. Small changes in the molecular structure of these fatty acids exert a great influence on activating different PPAR. Structural information, as well as ligand-binding assays and gene transactivation, has been employed in the designing of new dual-action drugs against diabetes and metabolic syndrome. These derivative molecules may also be utilized as anti-inflammatory and anticancer agents [100].

#### 2.4.2. PUFAs-Conjugated Linoleic Acid

Conjugated linoleic acid (CLA) is a class of geometric and positional conjugated dienoic isomers produced during biological or industrial hydrogenation of linoleic acid (C18:2, n-6, c9, and c12). The major dietary sources of CLA are ruminant meat, dairy products, and partially hydrogenated vegetable oils [101]. In fact, PUFAs and their metabolites, including CLA, are endogenous PPAR-*γ* ligands [52, 102]. CLA was found to be a potent naturally occurring agonist for PPAR-*γ* [103].

N-3 PUFAs (i.e., linoleic acid, DHA, and EPA) elicit potent anti-inflammatory and immunoregulatory properties either directly [104, 105] or following transcellular processing that results in the generation of hydroxy-containing n-3 PUFAs metabolites [106].

Recently, there has been extensive interest in the potential health benefits of dietary supplementation with CLA, including anti-carcinogenic and antitumorigenic effects [53], reduction in the risk of atherosclerosis, hypertension, and diabetes, improvement in food efficiency, promotion of energy metabolism, and an antiosteoporotic and positive effect on immune function [107]. Also, dietary-supplemented CLA is able to suppress cardiomyocyte hypertrophy through activation of PPAR-*α* and -*γ* [108]. Additionally, because, CLA profoundly reduces body fat stores in several species, including pig, rat, hamster, chicken, and mouse [109], it is popularly employed as a weight-loss management strategy [107, 110]. In addition, CLA has numerous additional reported benefits, including altering body composition, improving lipid profiles, modifying both innate and adaptive immune responses, and improving insulin resistance (reviewed in [111]).

However, CLA has been shown to exhibit some adverse effects [112], including reduction in insulin sensitivity in subjects with type 2 diabetes [113] and augmentation of the pre-existing insulin resistance [114, 115]. Additionally, long-term studies in rodents have demonstrated a lipodystrophic effect of CLA [116] that is associated with decreased plasma adiponectin and leptin levels and increased insulin resistance [117].

To overcome these adverse effects, a combination of CLA plus FO has been suggested for dietary supplementation during clinical trials in obese, insulin-resistant aging patients in order to prevent osteoporosis [118]. This could result in an excellent strategy in the management of fat-mass reduction and osteoporotic bone loss, circumventing CLA-induced hepatomegaly and insulin resistance, although additional studies in humans are still required before the use of CLA+FO as a dietary supplement to reduce obesity and osteoporosis in humans [118].

Two main mechanistic theories have been proposed to explain the immune-enhancing effects of dietary CLA: a PPAR-*γ*-dependent and a PPAR-*γ*-independent pathway [102]. *In vitro* studies have also indicated that the PPAR-*γ*-activating capabilities of CLA are cell type-dependent and isomer-specific [102], and *in vivo*, *PPAR-*γ** gene expression has been proposed as a mediator of CLA effects [119]. In support to this theory, it has been reported that dietary n-3 PUFAS (DHA and EPA) in a mutant mice could reduce hypertrophy and hyperplasia of fat cells *in vivo*. This inducible and reversible model of lipoatrophy was achieved by selective ablation of PPAR-*γ* in response to tamoxifen [120]. 

## 3. Phytochemicals

Phytochemicals are non-nutritive plant chemicals that possess protective or disease-preventive properties. They are nonessential nutrients, meaning that the human body does not require them for sustaining life.

### 3.1. Isoflavones and Flavonoids

Botanical compounds such as isoflavones may be agonists or activators of the “promiscuous” PPAR nuclear receptors regulating lipid metabolism in the cell [54, 121–123], and glucose tolerance [54]. Isoflavone intake is altering lipid metabolism in a manner consistent with activation of PPAR-*γ* and also via a PPAR-*γ*-independent mechanism [124], possibly mediated by PPAR-*γ* activation. In particular, genistein, a soy isoflavone, has been identified as a ligand of the PPAR-*γ* receptor [121].

In contrast, flavonoids such as quercetin inhibited the activation of all three isoforms of PPAR [55], but its metabolites upregulated PPAR-*γ* expression [125] and could alleviate hepatic fat accumulation [126]. In addition, chickens fed diets supplemented with quercetin appear to prevent some of the damage attributable to the dysregulated inflammatory responses associated with aging by reducing serum levels of TNF-*α* and NO [73]. Additionally, some phytoestrogens, including soy isoflavones, have revealed their potential for inducing hormone-dependent cancers (breast and endometrium), leading to safety concerns [127]. Thus, a maximum daily intake level for phytoestrogens has been suggested in several countries [128].

### 3.2. Lectins

Lectins form an unavoidable component of the vegetarian diet; yet, their effects on various tissues have not been extensively studied. Many of the reported studies have focused their attention on gastrointestinal tract or on immune system with respect to allergic responses. However, it is relevant to examine the effects of apparently nontoxic dietary lectins on animal tissues and humans regarding its adipogenic effect [129]. Two dietary lectins isolated from common foods usually consumed in an unprocessed form are Banana lectin (BL) and Garlic lectin (GL). Oral administration of these lectins resulted in an enhanced hematopoietic stem progenitor cell pool of mice [130]. In addition, both lectins, BL and GL, exert an adipogenic effect on these mesenchymal cells and significantly up-regulate PPAR-*γ*2 expression [129], an effect comparable to that mediated by insulin. Both of these lectins interact with the insulin receptor in this cell line and activate the Mitogen-activated protein kinase kinase (MEK)-dependent Extracellular signal-regulated kinase (ERK) pathway [130]. Therefore, it is possible to use specific subfragments or peptide sequences derived from these lectins to develop agonists or antagonists of insulin receptor-mediated signaling to further examine the *in vivo* effects of dietary lectins on tissue homeostasis [129].

### 3.3. Alliin and Allicin from Garlic (*Allium Sativum*)

Garlic has been used for 5,000 years, not only as a culinary spice, but also as a medicinal herb due to its antibacterial and anti-infectious properties. It has been utilized to treat a variety of health problems [131, 132] due to its high content of organosulfur compounds and its antioxidant activity.

The main active component in garlic is the S-allyl cysteine sulfoxide, commonly called alliin, which presents a cardioprotective effect in a model of myocardial infarct [56]. In addition, alliin reduce the levels of TNF-*α* in human-umbilical-vein endothelial cells and helps to decrease serum levels of glucose, insulin, triglycerides, and uric acid, as well as insulin resistance, when compared with fructose-fed rats [133, 134].

Another organosulfurated compound from garlic is 1,2-vinyldithiin (1,2-DT) [135], a lipophilic component found mainly in oily macerates of crushed garlic, arising from the degradation of allicin [136]. This 1,2-DT inhibits the differentiation and inflammation of the human preadipocyte *in vitro*, an effect that is mediated by a significant reduction in the expression of the following key major adipogenic-transcription factors: PPAR-*γ*2 and CCAAT-enhancer-binding protein CCAAT-enhancer-binding protein-alpha (C/EBP)-*α*, induced by 1,2-DT during human preadipocyte differentiation [137]. This suggests that the negative effect of 1,2-DT on preadipocyte differentiation could be mainly due to an inhibitory effect on PPAR-*γ*2 and on its target gene, lipoprotein lipase (*LPL*), regardless of the period of exposure in human primary preadipocytes [137]. As a consequence, 1,2-DT could constitute an original dietary supplement in the treatment of obesity and its associated pathologies by limiting the expansion and inflammation of human white adipose tissue.

On the other hand, another constituent derived from garlic, diallyl disulfide, was shown to accelerate the differentiation of 3T3-L1 cells via PPAR-*γ* activation [138]. In addition, our recent observations (unpublished) indicated that exposure of 3T3-L1 adipocytes to alliin is able to suppress LPS-evoked molecular inflammatory signals by causing a decrease in the messenger RNA (mRNA) expression level of pro-inflammatory genes (*IL-6*,* MCP-1*, and *Egr-1*) in this *in vitro *model of adipose tissue [139]. Therefore, more research continued to be needed to elucidate the complete molecular mechanism.

### 3.4. Curcumin, the Indian Spice Turmeric (*Zingiberaceae*)

Curcumin is an antioxidant, anti-inflammatory, active principal ingredient of the curry spice, turmeric. The compound is marketed as a dietary supplement [140] and has attracted interest as a cancer-preventive agent [57].

It is well known that curcumin prevents the onset of inflammation by inhibiting the activation of nuclear factor-kappa beta (NF-*κ*B), the production of TNF-*α*, interferon-gamma (IFN-*γ*), and NO, and the gene expression of inducible nitric oxide synthase (*iNOS*) [141–143]. It acts by transrepressing NF-*κ*B, activating protein-1, and the signal transducer and activator of transcription-1 [144–148].

Curcumin activates PPAR-*γ* in Moser cells, a human colon cancer cell line [145], and is able to suppress sepsis through PPAR-*γ* [146]. In addition, it increased PPAR and decreased *iNOS* gene expression in infected macrophages, as well as downregulated IFN-*γ* production by primed lymphocytes [147]. Curcumin action on PPAR could involve a curcumin-responsive element that resides in the *PPAR-*γ** gene regulatory region [148].

### 3.5. Resveratrol: A Phytoalexin from Grapes

Resveratrol is a naturally occurring polyphenolic compound with strong antioxidant properties and is found in abundance as a component of red wine and blackberries. Research has described several beneficial properties of this compound, including anti-carcinogenic, antiaging, neuroprotective, analgesic, antidiabetic, and antiobesity effects (reviewed in [149]).

Related with their antidiabetic and anti-obesity effects, PPAR-*γ* is one of the targets of resveratrol [58], which partially mediates its antiadipogenic and proapoptotic effects in 3T3-L1 adipocytes [150, 151] by preventing TNF-*α*-induced suppression of adiponectin expression [152]. Also, resveratrol induces changes in white adipose-tissue metabolism in rats [59] and up-regulates SIRT1, FOXO1, and adiponectin, down-regulating PPAR-*γ*1−3 mRNA expression in human visceral adipocytes [153]. In addition, resveratrol prevents the impairment of advanced glycosylation end-products (AGE) on macrophage lipid homeostasis by suppressing the receptor for AGE via PPAR-*γ* activation [154].

### 3.6. Triterpenes and Polysaccharides from the Medicinal Mushroom *Ganoderma lucidum*

A dietary supplement containing triterpenes and polysaccharides extracted from the medicinal mushroom *G. lucidum* is effective in inhibiting proliferation, invasive behavior, and angiogenesis in different cancer models and also as a treatment for atherosclerosis (reviewed in [155]). Very recently, a positive effect of *G. lucidum* on the metabolic syndrome has been investigated. Treatment of 3T3-L1 cells with *G. lucidum* extract significantly promoted adipocyte differentiation and adiponectin production in a dose-dependent manner [156]. This effect is suppressed by GW9662, a PPAR-*γ* inhibitor, suggesting the involvement of this receptor [156]. Also, certain *G. lucidum*-derived lanostane triterpenes are able to suppress PPAR-*γ* expression in 3T3-L1 cells [60], and a dietary supplement, ReishiMax, containing triterpenes and polysaccharides extracted from *G. lucidum* affects adipocyte differentiation and glucose uptake in 3T3-L1 cells [157] through decrease in the expression of PPAR-*γ*, sterol regulatory binding protein (SREBP)-1c, and C/EBP-*α*, which regulate the genes responsible for the synthesis and transport of fatty acids. Therefore, ReishiMax could inhibit adipocyte differentiation/lipid accumulation through the following: (a) down-regulation of the expression of transcription factors PPAR-*γ*, SREBP-1c and C/EBP-*α*, and (b) suppression of the expression of the genes responsible for lipid synthesis (*FAS*, *ACS1*), lipid transport (*FABP4*, *FATP1*), and lipid storage (perilipin, PLIN) [158].

## 4. Dietary Fiber

Several studies report interest in nondigestible carbohydrates, which are prone to fermentation by gut microbiota in the control of obesity and related metabolic disorders [158]. Carbohydrates showing a prebiotic effect have received special attention in this context, because they have been shown—mainly in experimental animal studies—to regulate food intake and weight gain, as well as metabolic disorders associated with obesity, such as liver steatosis, dyslipidemia, diabetes, and/or even hypertension [61, 158, 159].

### 4.1. Oligosaccharides

Fructooligosaccharides (FOS) and mannooligosaccharides are the most frequently encountered commercial forms of nondigestible carbohydrate polymers (prebiotics). These complex oligosaccharides contain 3–10 monosaccharide residues covalently linked through glycosidic bonds. Incubation of Caco-2 cells with both oligosaccharides induced PPAR-*γ*. PPAR-*γ* regulates PGlyRP3 expression and triggers oligosaccharide-induced anti-inflammatory effects [160]. Antagonizing PPAR-*γ* by culturing the cells with GW9662 for 24 h inhibited oligosaccharide-induced PGlyRP3 production and the anti-inflammatory effect of the oligosaccharides. Therefore, oligosaccharides may exert an anti-inflammatory effect by inducing PPAR-*γ*, which regulates anti-inflammatory PGlyRP3 [160]. Consequently, prebiotic oligosaccharides may modulate the inflammatory state by a direct anti-inflammatory effect. This effect appears to be PPAR-*γ* dependent and at least in part depends on PGlyRP3 expression.

### 4.2. Neolignans from *Magnolia officinalis*

The term neolignan was defined by Gottlieb in 1978 [161] as including the lignans and also related compounds in which the two C_6_C_3_ units are joined by other bonds (e.g., 3-3′ instead of 8-8′). In a review of natural resins, Haworth [162] proposed that the class of compounds derived from two C_6_C_3_ *β*,*β*′-linked units should be called lignans (his original spelling was lignane, but the “e” was deleted in subsequent publications).

Recently, in a computer-assisted search for PPAR-*γ* agonists by three-dimensional (3D) structure homology, some neolignans emerge as strong candidates. Two of these, dieugenol and tetrahydrodieugenol, can be isolated from *Magnolia officinalis* Rehd. et Wils. bark with the use of different chromatographic methods [163]. These naturally derived compounds act as partial PPAR-*γ* agonists; both exhibited higher affinity for PPAR-*γ* than for the clinically used agonist pioglitazone (Actos) and were identified as selective activators of PPAR-*γ*, but not of PPAR-*α* or of PPAR-*β*/*δ*. In addition, these compounds induced adipocyte differentiation in 3T3-L1 cells in a PPAR-*γ*-dependent manner. The activation pattern exhibited from 1 and 2 renders them highly interesting, novel PPAR-*γ* agonists that have the potential to be explored further which may lead to the development of novel pharmaceuticals or dietary supplements [163].

## 5. Probiotics

As defined in the previous section, prebiotics are nondigestible food components that beneficially affect the human body through modulation of the intestinal microbiota by selectively stimulating the growth and/or activity of some bacteria species in the colon, which are denominated “probiotics” [161, 164].

Probiotics are unicellular endosymbionts (microbes), such as lactic acid bacteria, bifidobacteria, yeast, and bacilli, which are thought to be beneficial to the host organism. The beneficial effects of probiotics extend outside of the intestine and have been shown to exert beneficial effects in obesity; nonalcoholic steatohepatitis (NASH) and diabetes, despite the molecular and cellular mechanisms mediating these effects, have not been completely elucidated [165].

### 5.1. *Saccharomyces cerevisiae*

The yeast *Saccharomyces cerevisiae* variety *boulardii* (Sb) has been prescribed over the past 30 years for prophylaxis and treatment of diarrheal diseases caused by bacteria and has been shown to provide intestinal protection against various enteric pathogens [166]. Indeed, *Saccharomyces boulardii* protected the host through multiple mechanisms such as inhibition of pathogen adhesion [167], neutralization of bacterial virulence factors [168], maintenance of epithelial barrier integrity [62], a decrease in pathogen-associated inflammation [161], and stimulation of the immune system [63]. This yeast has been shown to modulate pro-inflammatory signaling pathways leading to the inhibition of mitogen-activated protein kinases (MAPK) and NF-*κ*B activation in intestinal epithelial cells (IEC) [63, 169, 170].

The yeast Sc (strain CNCM I-3856) modulates transcript and protein expressions involved in inflammation, recruitment, and activation of immune cells in differentiated porcine intestinal epithelial IPEC-1 cells. Viable Sc inhibits enterotoxigenic *Escherichia coli-*(ETEC-) induced expression of pro-inflammatory transcripts (IL-6, IL-8, CCL20, CXCL2, and CXCL10) and proteins (IL-6 and IL-8). This inhibition was associated with a decrease of ERK1/2 and p38 MAPK phosphorylation, agglutination of ETEC by Sc, and an increase in the anti-inflammatory PPAR-*γ* mRNA level [171] to mediate anti-inflammatory effects.

*S. boulardii* upregulated PPAR-*γ* expression in human colonocytes and in HT-29 colonic epithelial cells [172] and raised the transcription of this receptor in the colon of animals exposed to TNBS ([172], and reviewed in [173]). Also, *S. boulardii* inhibited TNF-*α*-mediated regulation of PPAR-*γ* and IL-8 by blocking activation of NF-*κ*B [174].

### 5.2. *Escherichia coli*

The *Escherichia coli* strain Nissle 1917 (EcN) was isolated by A. Nissle in 1917 from the feces of a soldier who was nearly the only one who did not develop enterocolitis during the war on the Balkan peninsula [175]. At present, this bacterium is contained in a probiotic drug called Mutaflor, whose efficacy against inflammatory states in lower gastrointestinal tract has been demonstrated in numerous trials (reviewed in [176]). EcN draw out a number of favorable effects in the gut, including enhancement of their barrier function, a modulator effect on its motility, and a reduction in the formation and secretion of pro-inflammatory cytokines; it also exhibits an antagonistic activity against pathogenic bacteria in the gastrointestinal tract, induces immunomodulatory effects, and provide support of colonocyte metabolism (reviewed in [177]).

The gastroprotective activity of EcN could be at least partially attributed to modulation of PPAR expression. In a model of gastric mucosa exposed to stress, significant up-regulation of PPAR-*γ* at the protein level has been reported [178]; in this case, pretreatment with EcN attenuated the acute gastric mucosal lesions induced by stress through anti-inflammatory actions, induction of protective factors such as ghrelin and HSP70 synthesis in the gastric mucosa, and the enhancement of gastric microcirculation. These beneficial effects were accompanied by the decrease in PPAR-*γ* expression [178].

### 5.3. Compound VSL3

Compound VSL3 is a biotherapeutic agent (VSL Pharmaceuticals) formulated with of eight strains of bacteria (*Lactobacillus acidophilus *MB 443, *Lactobacillus delbrueckii* subsp. *bulgaricus* MB 453, *Lactobacillus casei* MB 451, *Lactobacillus plantarum* MB 452, *Bifidobacterium longum* Y10, *Bifidobacterium infantis* Y1, *Bifidobacterium breve* Y8, and *Streptococcus salivarius* subsp. *thermophilus* MB 455).

This probiotic intervention results in a site-specific reduction of inflammatory pathways with increased expression of mediators involved in PPAR signaling [179]. Its administration attenuated the development of signs and symptoms of colitis, reduced colonic expression of TNF-*α*, Interleukin (IL)-6, and IFN-*γ*, and reserved colonic down-regulation of PPAR-*γ*, PXR, and FXR caused by a TNBS model of induced colitis [24].

Mesenteric adipose tissue from rodent colitis (TNBS-treated animals) and Crohn's disease is metabolically active and shows inflammation-driven regulation of PPAR-*γ*, FXR, leptin, and adiponectin. The VSL3 probiotic prevents and corrects this inflammation-driven metabolic dysfunction [24]. VSL3 converts linoleic acid into CLA, *in vitro* and *in vivo*, inducing the up-regulation of PPAR-*γ*, a reduction in cancer cell viability, and the induction of apoptosis [180].

## 6. PPAR-Gamma-Related Diseases That Can Be Complementarily Treated with Nutraceutics

Obesity has led to alarming increases in the incidence of many chronic diseases, including type 2 diabetes and cardiovascular disease. Because overnutrition leads to obesity, manipulation of the dietary nutrient content is a logical means of alleviating this problem. In chronic disorders such as obesity and diabetes, mesenteric adipose tissue (MAT) hypertrophy is governed by the activation of PPAR family of nuclear receptors, mainly the *α* and *γ* forms, FXR, liver-X receptor (LXR), and adipokines, which are well identified targets for medical interventions. In contrast, it remains unknown whether MAT could be modulated by pharmacological interventions in Crohn's disease [181].

The commensal gut microbiota has profound effects on the physiology of the host [182]. The intestinal microbiota may be exerting effects beyond the intestine and patients with chronic inflammatory disorders such as obesity and type I diabetes display an altered gut flora that may have a pathogenetic readout on the phenotype of these disorders [182–184].

Thirty years ago, it was largely appreciated that circulating levels of xeno- and endobiotics including bile acids, lipids, and metabolism intermediates are regulated by gut microbiota [185–187]. Thus, activation of PPAR-*α* and -*γ*, FXR, and LXR by lipid mediators, bile acids, and oxysterols not only modulates lipid/cholesterol metabolism, but also provides counter-regulatory signals for innate immunity cells [7, 8, 24, 188–190].

The immune system highly depends on a satisfactory supply of nutrients to function. Few studies have considered the immunomodulatory effects of oligosaccharides in humans and experimental animal models. Daily administration of different oligosaccharides significantly reduced damage associated with Crohn's disease, ulcerative colitis, and experimental colitis in rats [191–194].

### 6.1. Inflammatory Bowel Diseases (IBD)

Inflammatory bowel disease (IBD) is a chronic, recurring immuno-inflammatory illness afflicting 0.1–24.5/100,000 people worldwide with two clinical manifestations: Crohn's disease (CD) and Ulcerative colitis (UC) [195].

A growing body of evidence supports the notion that a mutual inhibition between pro-inflammatory mediators and nuclear receptors does exist in IBD [196, 197]. Evidence also suggests the potential therapeutic role and beneficial immunomodulatory effects of some commensal gut microbiota, such as probiotics, in the prevention or suitable treatment of gastrointestinal tract diseases [198] and mild-to-moderate IBD by inducing immune homeostasis.

Clearly, PPAR-*γ* represents a novel therapeutic target useful in reducing intestinal inflammation for the treatment of IBD [22, 199]. Consistent with this view, Mencarelli et al. reported that acute TNBS colitis causes a robust decrease in the expression of several nuclear receptors including PPAR-*γ*, PXR, and FXR, and that these changes were antagonized by VSL#3 cotreatment [24]. In addition, developing nutritional interventions against IBD, such as diets supplemented with CLA and n-3 PUFAs, is relevant [200] because this has been reported to ameliorate intestinal inflammation in animal models of IBD [201], an effect possibly related with bacterial recognition sites in Toll-like receptors (TLR) [202].

### 6.2. Crohn's Disease

This is a chronic and progressive inflammatory disorder of gastrointestinal tract [24]. Adipose tissue is increasingly identified as a major endocrine organ from which either metabolic or inflammatory signals propagate systemically, potentially modulating clinical features of Crohn's disease. Patients with Crohn's disease accumulate adipokine-releasing intra-abdominal fat from disease onset [203, 204], indicating that expansion of mesenteric fat depots may be an important source of inflammatory mediators in IBD [205]. An abnormal expression of PPAR-*γ* has been reported in the MAT of patients with Crohn's disease [203].

Present results and previous data demonstrate that FXR exercises anti-inflammatory activity in rodent models of colitis [185] while promoting a less activated phenotype in adipose tissue [10], suggesting a potential therapeutic role for ligands of this nuclear receptor in the treatment of inflammation-driven activation of adipose tissue in Crohn's disease [24].

In conclusion, it has been shown that colonic inflammation regulates the expression of several nuclear receptors in MAT in a model of colitis and in patients with Crohn's disease. MAT activation could contribute to inflammation-driven immune and metabolic dysfunction in these patients by generating a subset of pro-inflammatory mediators and modulating the expression of several nuclear receptors. Whether PPAR-*γ* expression is causal to susceptibility and/or pivotal in modulating adaptive immunity remains under investigation. These effects are counter-regulated by changing the composition of enteric flora with a probiotic [24].

### 6.3. Cardiac Disease

Activation of PPAR-*α* and -*γ* by CLA prevents cardiac hypertrophy through activation of the diacyl glycerol kinase-zeta (DGK-*ζ*) and subsequent inhibition of the protein kinase C-epsilon (PKC-*ε*) pathway. These findings provide novel support for the role of PUFAs as cardioprotective dietary elements. At a time when the prevention of hypertrophy is viewed as a promising therapeutic target [206] and 44% of patients with heart failure resort to nutrition-based therapies [207], this study may have important implications for effective nutritional intervention toward the prevention of cardiac disease. Finally, evidence from a clinical study indicate that dietary intake of omega-3 fatty acids, particularly the DHA and EPA found in fatty fish or fish-oil supplements, reduces the risk of CVD [208].

## 7. Concluding Remarks

Despite the great potential benefits that nutraceutics are able to provide, one must be careful when consuming botanical and dietary supplements with anti-inflammatory and antioxidant properties when one is at risk for microbial infections. *In vivo*, the outcome of an infection is determined by a balance between means of host-immune defense versus those of the parasite, and antioxidants and anti-inflammatories may weaken immune defense and exacerbate the infection. Furthermore, the high prevalence of dietary supplements does not ensure that the nutrient intake of supplements would be the same among all of their consumers. The nutrient intake from dietary supplements varies in terms of the composition of the supplements. Therefore, it is urgent to meet nutrient needs by consuming foods that provide a well-balanced, nutrient-dense diet.

It is also important to engage in different types of evidence in support of the health benefits of natural products. Epidemiological information may offer the first suggestion that certain natural products, in the diets of specific populations, may exert an influence on the course of chronic diseases, such as obesity and some of its concomitant complications, such as cardiovascular diseases, diabetes, and cancer. Although the intake of certain dietary compounds indicate differences in the prevalence of some pathologies in specific groups, it does not prove that supplementation with these compounds could change the course of the pathologies. Genetic and environmental factors may all contribute to the final effects observed in a studied population.

In this paper, we carried out a detailed approach on the mechanistic and molecular interaction of nutraceutics and PPAR-*γ*, which has been identified as one of the major regulators of adipogenesis at a cellular level and a master regulator of energetic homeostasis. Because the use of synthetic PPAR-*γ* agonists has been associated with an increased risk of cardiovascular ischemic events, natural PPAR-*γ* ligands have been shown to ameliorate obesity-related disorders and certain inflammatory diseases. Some of these agonists could activate both PPAR-*α* and PPAR-*γ* (dual PPAR-*α*/PPAR-*γ* agonists), which might be even more effective. However, additional, extensive research of nutraceutics and their potential ability to modulate PPAR-*γ* in strengthening the inflammatory response network requires further study in the future. Unfortunately, there continue to be few clinical trials on these compounds and some evidences lack the molecular mechanisms. The group of evidences indicates that the effect of molecular interactions will also depend on the model studied in the laboratory. *In vivo* and *in vitro* assays have documented the biological effects on adipogenesis, inflammation, carcinogenesis, and so forth. However, additional studies should be conducted in PPAR-*γ* conditional mice in which the effects of nutraceutics on adipose tissues the hyperplasia and hypertrophy, adipogenetic genes modulation, and inflammatory biomarkers can be directly observed and studied.

In conclusion, although more experimental work is required to evaluate their full potential in humans, especially in terms of safety, PPAR natural agonists nonetheless represent a promising strategy for mitigating obesity-related disorders and some inflammatory diseases, reducing the side effects exhibited by the commonly used pharmacological drugs. However, more randomized controlled trials are needed for nutraceutics that, in agreement with epidemiological and mechanistic evidence assays, could be good candidates for or against a specific pathology. Additionally, surprising results of increased disease risk with the consumption of some natural products have been found. Therefore, unless safety profiles for nutraceutical supplements in humans are available, caution should be used in their long-term use as PPAR-*γ* modulators.

## Figures and Tables

**Table 1:** Functional foods classification, some sources, and examples of bioactive substances.

Functional food	Bioactive component (nutraceutic)		Source (s)
Micronutrients	Vitamins	Retinol (vitamin A) *α*-tocopherol (vitamin E) Calciferol (vitamin D3)	Walnuts, almonds, hazelnuts, spinach, fish oil
	Polyunsaturated fatty acids (PUFAs)	Omega 3 Fatty acids: eicosapentaenoic acid (EPA) docosahexaenoic acid (DHA)	Salmon, tuna and others fish oils

Nonnutrients Phytochemicals	Carotenoids	Beta-carotene luteín, zeaxanthin lycopene	Carrots, pumpkin, collards, kale, spinach, tomatoes, watermelon
	Phenolic acid derivatives	Caffeic acid Ferulic acid Gallic acid Curcumin	Coffee, pears, apples, corn, curcumin, vanilla
	Flavonoids	Flavonols (quercetin) Isoflavones Coumarins Anthocyanidines Stilbenes (resveratrol)	Berries, cherries, red grapes, tea, cocoa, apples, citrus fruits, onion, broccoli, cranberries, strawberries, soybeans
	Sulfides/thiols	Diallyl sulfide S-allyl cysteine sulfoxide 1,2-viniyldithiin	Garlic, onions, banana, cruciferous vegetables
	Dietary fiber (prebiotic)	Fructooligosaccharides Neoglicans	Whole grains, onions, chicory, agave, some fruits

Probiotics	PUFAs induction	*Saccharomyces cerevisiae* (var. *boulardii*) Bifidobacteria and *Lactobacillus* genus *Escherichia coli* strain Nissle1917 (EcN) Compound VSL3	Certain yogurts and other cultured dairy and no-dairy applications

**Table 2:** Modulation of PPAR-*γ* by nutraceutics.

Bioactive (nutraceutic)	Effect on PPAR-*γ*	Ref.
Retinaldehyde	Inhibit PPAR-*γ* activity in adipocyte cell cultures and mouse models	[45]
*β*-apo-149-carotenal	Inhibit PPAR-*γ* activity and adipogenesis in adipocyte cell culture	[46]
*α*- and *γ*-tocopherol	Activate PPAR-*γ* expression in colon cancer cells	[47, 48]
Retinoic acid and 1,25-dihydroxy vitamin D3	Inhibited adipocyte differentiation of 3T3-L1 preadipocytes by repressing the upregulated protein expression of PPAR-*γ*2	[49]
N3 fatty acids from fish oil	Increases adiponectin level and upregulates PPAR-*γ*	[50, 51]
Linoleic acid	Agonist for PPAR-*γ* Activation of PPAR-*α* and -*γ*	[52] [53]
Quercetin	Inhibited activation of all three isoforms of PPAR	[54]
Banana lectin and garlic lectin	Exert an adipogenic effect on mesenchymal cells and upregulate PPAR-*γ*2 expression	[55]
1,2-vinyldithiin (1,2-DT) (from garlic)	Inhibits differentiation and inflammation of human preadipocyte *in vitro* by a reduction in expression of PPAR-*γ*2	[56]
Curcumin	Activates PPAR-*γ* in colon cancer cell line	[57]
Resveratrol	Downregulates PPAR-*γ* 1–3 mRNA expression in human visceral adipocytes	[58]
Lanostane triterpenes	Suppress PPAR-*γ* expression in 3T3-L1 cells	[59]
Fructooligosaccharides and mannooligosaccharides	Induced PPAR-*γ* in Caco-2 cells	[60]
Neolignans	PPAR-*γ* agonists	[61]
*S. boulardii *	Upregulated PPAR-*γ* expression in human colonocytes and in HT-29 colonic epithelial cells	[62]
	Inhibited TNF-*α*-mediated regulation of PPAR-*γ*	[63]

PPAR: Peroxisome proliferation-activated receptor; PPAR-*γ*: PPAR-gamma; *S. boulardii*:* Saccharomyces boulardii*.

## Abbreviations

PPAR: Peroxisome proliferator-activated receptor
NASH: Nonalcoholic steatohepatitis
RXR: Retinoid-X receptor
FXR: Farnesoid-X receptor
LXR: Liver–x-receptor
FO: Fish oil
LPL: Lipoptrotein lipase
NF-*κ*B: Nuclear factor-*κ*B
iNOS: Inducible nitric oxide synthase
CLA: Conjugated linoleic acid
N-3 PUFAs: Polyunsaturated fatty acids
EPA: Eicosapentaenoic acid
DHA: Docosahexaenoic acid
BC: *β*-carotene
RA: Retinoic acid
BL: Banana lectin
GL: Garlic lectin
FOS: Fructooligosaccharides
MAT: Mesenteric adipose tissue
IBD: Inflammatory bowel disease
CD: Crohn's disease
UC: Ulcerative colitis.
